# Study on Microwave Absorption Performance Enhancement of Metamaterial/Honeycomb Sandwich Composites in the Low Frequency Band

**DOI:** 10.3390/polym14071424

**Published:** 2022-03-31

**Authors:** Songming Li, Hao Huang, Sibao Wu, Jiafu Wang, Haijun Lu, Liying Xing

**Affiliations:** 1 Composite Technology Center, AVIC Beijing Aeronautical Manufacturing Technology Research Institute, Beijing 101300, China; lisongming860206@126.com (S.L.); thu_huanghao@163.com (H.H.); wusibao0507@163.com (S.W.); haijunlu_hjl@163.com (H.L.); 2 National Key Laboratory of Advanced Composites, AECC Beijing Institute of Aeronautical Materials, Beijing 100095, China; 3 Department of Basic Sciences, Air Force Engineering University, Xi’an 710051, China; wangjiafu1981@126.com

**Keywords:** honeycomb sandwich composites, metamaterial, radar stealth, microwave absorbing material, low frequency

## Abstract

With the rapid development of electronic technology and modern radar detection system, there is increasingly urgent demand for microwave absorbing composites working efficiently in the low frequency range (e.g., 1–2 GHz). In this work, a type of metamaterial/honeycomb sandwich composite (MHSC) was proposed and fabricated, which exhibited a light weight structure and excellent wave-absorbing performance in the low frequency band. The relationship between the wave-absorbing properties and the design parameters of the composite, such as the thickness of the wave-transmitting skin, the thickness and dielectric properties of the wave-absorbing honeycomb, was systematically investigated. The electromagnetic coupling interference between the honeycomb absorber and metamaterial resonator proved to be a crucial factor that affects synergistic wave-absorbing performance in the low-frequency band. Under the rational design, the incorporation of subwavelength-sized phase-gradient metamaterial units in the composite can significantly improve low-frequency wave-absorbing performance for greater than 5 dB (an increment larger than 100%); and the obtained MHSC exhibits averaged reflectivity (*R*_a_) less than −10 dB in the low frequency band of 1–2 GHz as well as outstanding performance (*R*_a_ < −14.6 dB) over an extremely wide frequency range (1–18 GHz). The MHSC reported in this study could be a promising candidate for the key material in high-performance radar stealth and other related applications.

## 1. Introduction

In recent years, microwave-absorbing materials (MAMs) have attracted much attention in view of their practical applications in electromagnetic shielding, camouflage, radar stealth and other advanced technologies for both commercial and military purposes [1,2,3,4,5,6,7,8]. Compared with other types of MAMs (e.g., wave-absorbing coating), structural wave-absorbing composites show comprehensive superiorities, including high mechanical strength, heat resistance and broadband absorption ability [9]. They exhibit dual functions for load-bearing and wave-absorbing in an integral structure and are widely used in radar stealth equipment [10]. Nowadays, with the rapid evolution of electronic technology, modern radar systems can effectively detect a target within a broad frequency band (e.g., 1–18 GHz). In particular, low frequency early warning radar (1–2 GHz) has become the most predominant threat for conventional military planes and even stealth aircraft [11,12,13,14]. As a result, there is an increasingly urgent demand for structural wave-absorbing composites having light weight structure, broadband absorption ability, and high performance low-frequency radar stealth function at the same time [14]. This presents a great challenge in the design and fabrication of such material systems within the bounds of current knowledge.

Honeycomb sandwich composites (HSCs) are widely used in aeronautical structures and automobiles due to their advantages, such as being light weight, having high stiffness-to-mass ratio, good heat-insulation performance and so on [15]. By coating or filling hexagonal honeycomb cores with lossy agents, an HSC can become an efficient MAM [16]. The wave-absorbing performance of an HSC is affected by a series of design parameters, such as thickness, core size and orientation of the honeycomb [17,18,19], coating thickness and dielectric properties of the lossy agents [20,21,22], and even the fabrication/molding process [23]. Vast work and progress have been made by using varied lossy agents, including dielectric materials (e.g., carbon black, graphene and multi-wall carbon nanotube) and magnetic materials (e.g., carbonyl iron, ferrite and nickel metal) to fabricate wave-absorbing HSCs with low reflectivity and high absorptivity [17,21,24,25,26,27,28,29,30,31]. Nevertheless, early studies mainly focused on enhancing the wave-absorbing properties of HSCs in a relatively high frequency band within 2–18 GHz (mostly 8–18 GHz). By contrast, there is still a lack of effective methodologies to realize satisfactory radar stealth performance (averaged reflectivity less than −10 dB) in the low frequency band of 1–2 GHz for conventional HSC. Although increasing the thickness of honeycomb, using a high content of lossy agents, or employing magnetic materials with high complex permeability may be helpful in improving low-frequency wave-absorbing performance [14,32], this will inevitably result in drastic weight increase for the total structure, which is infeasible for practical applications. Consequently, it is necessary to introduce new technical approaches and mechanisms to fundamentally solve this problem.

Metamaterials are kinds of artificial materials which consist of subwavelength structural units and possess many peculiar electromagnetic properties unattainable with natural materials [33,34,35]. By arranging resonant units in a periodic, quasi-periodic or disordered/coded manner, the design of metamaterials can exhibit artificially customized permittivity and permeability following predetermined spatial distribution, resulting in full control of the amplitude, phase, polarization, propagation and dispersion of the electromagnetic wave [36,37,38]. Compared with the traditional microwave absorber, metamaterial not only provides new perspectives on the wave-absorbing mechanism but also offers more practical approaches to realize high performance radar stealth with a light-weight structure [39,40,41]. Among metamaterials, phase-gradient metasurface (PGM), which is based on generalized laws of reflection and refraction (Snell’s Law), is a promising candidate to solve the bottleneck problem of traditional wave-absorbing material in the low frequency region [42,43,44,45]. Through the abnormal reflection properties of PGM, the main lobe of a reflected radar wave can be deviated in a non-threatening direction to reduce radar cross section (RCS); meanwhile, due to abnormal refraction, the effective thickness of the absorber below PGM is significantly increased to enhance the energy dissipation of the electromagnetic wave [46,47]. Although it is expected that the incorporation of metamaterial can possibly improve absorption efficiencies, or widen the absorption band of conventional MAMs, at the current stage, there is still lack of study concerning the fabrication and wave-absorbing performance of honeycomb sandwich composites loaded with metamaterials [48,49]. Huang et al. [48] proposed a type of wave-absorbing honeycomb containing “H-shaped” metamaterial, where the inner wall of wave-transmitting honeycomb cores was first posted with metal units and then impregnated with wave-absorbing resin. The reflectivity of the metamaterial-loaded honeycomb is less than −5 dB in the frequency band of 2~18 GHz. However, electromagnetic interaction and coupling interference between the metamaterial units and honeycomb absorber, especially in the low-frequency microwave region, is still ambiguous. It is of vital significance and urgent demand to investigate the synergistic/conflict effect of the two absorbing mechanisms and to optimize design parameters to the composites, in order to achieve satisfactory low-frequency and broadband wave-absorbing performance within a light-weight structure.

In this work, we reported on a type of metamaterial/honeycomb sandwich composite (MHSC) which exhibited excellent microwave absorption performance in the low frequency band of 1–2 GHz. The metamaterial units designed, following the phase-gradient principle, were incorporated into MHSC via a pattern-transfer method and molded together with the composite. The influences of the design parameters (including the thickness of skin, and thickness and dielectric properties of honeycomb) on the low-frequency wave-absorbing properties of the composites were systematically investigated, and the related mechanisms were elucidated. It was demonstrated that coupling interference between the honeycomb absorber and metamaterial resonator was the most crucial factor that affected their synergistic wave-absorbing performance in the low-frequency band. On the basis of the above understanding, an optimized radar stealth performance with averaged reflectivity (*R*_a_) less than −10 dB in the band of 1–2 GHz was successfully obtained for MHSC, while there was no significant weight increase compared with the original HSC. On this occasion, the MHSC also exhibited excellent radar stealth performance (*R*_a_ < −14.6 dB) over an extremely wide frequency range of 1–18 GHz, which shows great opportunities for real applications.

## 2. Materials and Methods

### 2.1. Materials

Quartz fiber/bismaleimides wave-transmitting prepregs (QW280/5429), wave-absorbing honeycomb (SKuF), wave-transmitting Nomex honeycomb (NH-1-2.7-48) and carbon fiber/bismaleimides prepregs (ZT7H/5429) were purchased from AVIC Composite Corporation Ltd., Beijing, China. The poly(ether-ether-ketone) (PEEK) film with the thickness of 10–15 μm was fabricated by solution casting, following the literature [50]. Epoxy glue film (J-116) was purchased from the Institute of Petrochemistry, Heilongjiang Academy of Sciences, Harbin, China. All the reagents and materials were purchased from commercial sources and used as received without further purification.

### 2.2. Fabrication of MHSC

**Figure 1:**
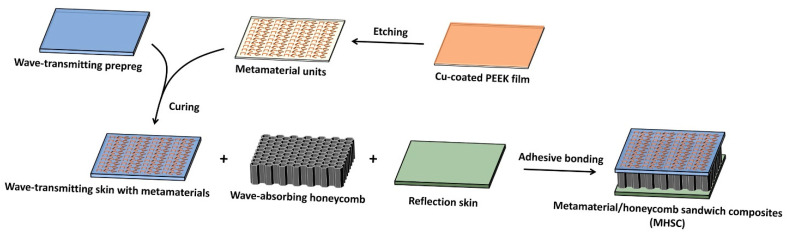
Schematic illustration about the fabrication of the metamaterial/honeycomb sandwich composites (MHSCs).

The fabrication process of the metamaterial/honeycomb sandwich composites (MHSCs) is schematically shown in Figure 1. First, the PEEK film was plated with a thin copper layer by magnetron sputtering, and the periodic metal units were then fabricated by wet-etching, according to the designed patterns, to obtain the metamaterial layer. The wave-transmitting skin and the reflection skin were prepared by the autoclave vacuum bag molding method. To fabricate the wave-transmitting skin, several layers of the QW280/5429 prepregs were stacked together with the metamaterial layer, which were cured at 180 °C for 3 h, followed by post-curing at 200 °C for 5 h. During the curing process, a vacuum and a pressure of 0.7 MPa was applied. A photograph of the fabricated wave-transmitting skin containing metamaterial is shown in Figure S1 in the Supplementary Materials, where the dark patterns are the periodic metal units. The reflection skin was prepared by curing ZT7H/5429 prepregs using the same procedure. Finally, the wave-transmitting skin, containing metamaterial units, the wave-absorbing honeycomb and the reflection skin were bonded together by J-116 glue film at a temperature of 180 °C under a pressure of 0.3 MPa for 2 h [51], by which means the MHSCs were successfully fabricated. To investigate the effect of metamaterial on wave-absorbing properties, conventional HSC was also fabricated, which uses the same procedure as above, except that the wave-transmitting prepregs were cured alone without adding the metamaterial layer.

### 2.3. Characterization

The dielectric property (i.e., the complex relative permittivity, *ε*_r_ = *ε*_r_′ + i*ε*_r_″) of the wave-absorbing honeycomb was measured by the free space method in a frequency range of 1–2 GHz. Two spot-focusing horn lens antennae (transmitting and receiving antennae) were placed face-to-face at a distance of twice the focal length. A honeycomb sample with an area of 600 mm × 600 mm was mounted on the focal plane of both the antennae. The *S*-parameters (Scattering parameters) of the sample in free space was measured by a vector network analyzer (Keysight PNA-L). The complex relative permittivity was calculated from the input reflection scattering parameter (*S*_11_) and the forward transmission scattering parameter (*S*_21_), by using the Nicholson-Ross algorithm [52]. The reflectivity (*R*, unit: dB) of the MHSC and HSC was measured by the NRL-arc method in a frequency range of 1–2 GHz. The transmitting antenna and receiving antenna, connected to a vector network analyzer (Keysight PNA-L), were mounted on top of the sample at normal incidence. Wave-absorbing pyramids were placed beneath the sample to suppress the reflection of the background. The value of *R* was calculated using the equation *R* = 10log(*P*/*P*_m_), where *P* and *P*_m_ are respectively the reflection power of the composite with an area of 600 mm × 600 mm and the reflection power of the metal sheet with the same dimensions. All the tests were conducted at a temperature of 25 °C.

## 3. Results and Discussion

### 3.1. Wave-Absorbing Properties of HSC

Honeycomb sandwich composite (HSC) is generally composed of a wave-transmitting skin, a wave-absorbing honeycomb and a reflection skin. The wave-absorbing mechanism of HSC is schematically illustrated in Figure S2 in the Supplementary Materials. When the electromagnetic wave is incident onto the HSC, it will first interact with the wave-transmitting skin. The material of this layer often exhibits low dielectric constant and low loss tangent (tan *δ* = *ε*_r_″/*ε*_r_′) [53,54], in order to realize good impedance matching with the free space and to decrease reflection loss (increase transmittance) on the surface of the HSC. With good performance of the wave-transmitting skin, the electromagnetic wave energy can enter into the HSC as much as possible. As the electromagnetic wave enters into the wave-absorbing honeycomb, it will be scattered in the periodic hexagonal structure and attenuated by the wave-absorbing resin adhering to the honeycomb surface (Figure S2). To obtain rapid and sufficient attenuation of the electromagnetic wave, the honeycomb should have relatively large dielectric loss and sufficient thickness. Meanwhile, impedance matching between the skin layer and the honeycomb is also important to suppress reflection on the interface and realize the maximum absorption ratio of the radar waves. The reflection skin consists of carbon fiber reinforced bismaleimides resin, which is the bottom layer of the HSC. Due to the high conductivity of carbon fiber, this layer can be regarded as a reflection layer and shields electromagnetic signals on the back side of the HSC. According to the above analysis, it is realized that the rational design of the wave-transmitting skin and the wave-absorbing honeycomb are very important for wave-absorbing performance of the HSC. The effects of thickness of wave-transmitting skin, dielectric properties of wave-absorbing honeycomb and thickness of wave-absorbing honeycomb on wave absorbing properties of the HSC are presented as follows.

#### 3.1.1. Effect of the Thickness of the Wave-Transmitting Skin

**Figure 2:**
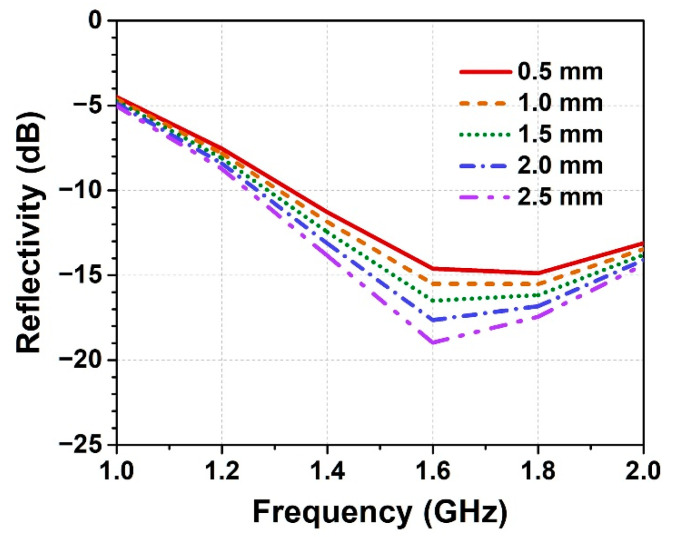
Reflectivity of the honeycomb sandwich composites (HSCs) with different thicknesses of the wave-transmitting skin.

For wave-absorbing HSC, the wave-transmitting skin is the first layer of material that interacts with the incident electromagnetic wave. The impedance matching between the skin and free space determines direct reflection on the surface and affects the integral wave-absorbing performance of HSC. Herein, the quartz fiber (*ε*_r_′ = 3.7, tan *δ* = 0.0005) and bismaleimides (*ε*_r_′ = 3.3, tan *δ* = 0.01) with low dielectric constant and low loss tangent were respectively selected as the reinforcing fiber and resin matrix of the wave-transmitting skin. Under the same composition of material, the thickness of the skin layer is the main factor in influencing transmittance of the electromagnetic wave and wave-absorbing performance. Wave-transmitting skins with different thicknesses were fabricated by adjusting the number of plies of the stacked prepregs in the curing step. Figure 2 shows the reflectivity of the HSC with different thicknesses (0.5–2.5 mm) of the wave-transmitting skin in the low frequency range (1–2 GHz), where the same type and thickness of honeycomb is used. The results illustrate that the wave-absorbing performance of HSC is slightly enhanced by increasing the thickness of the wave-transmitting skin. This phenomenon is ascribed to the long wavelength of the electromagnetic wave in this frequency band, which can sufficiently penetrate through the skin layer with negligible dielectric loss. However, increasing the thickness of the wave-transmitting skin can also weaken wave-absorbing properties in the high frequency range (e.g., 12–18 GHz) [13], and increase the total weight of the HSC structure, which works against the demand for wide-band radar stealth using light-weight material. To balance wave-absorbing performance, weight and mechanical property of the HSC, a moderate thickness (1 mm) of wave-transmitting skin is selected and used in the following sections.

#### 3.1.2. Effect of Dielectric Properties of Wave-Absorbing Honeycomb

**Figure 3:**
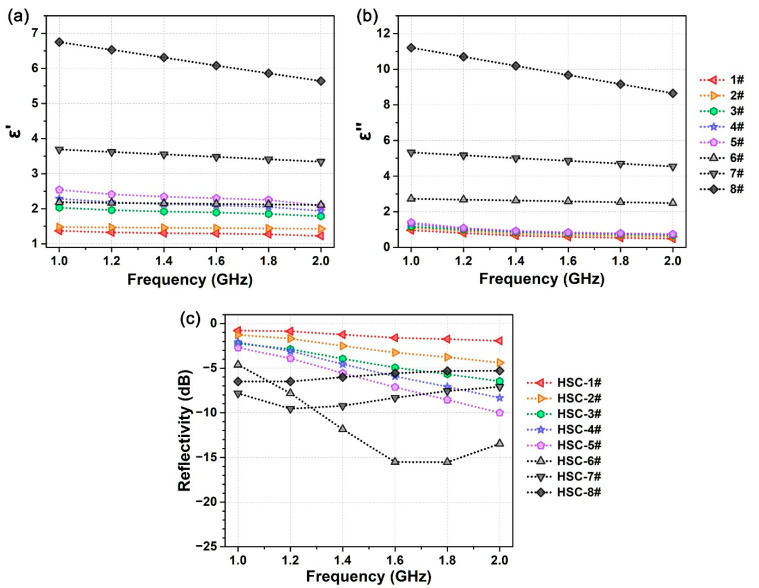
(**a**) Real part and (**b**) imaginary part of the complex relative permittivity (*ε*_r_′ + i*ε*_r_″) of different wave-absorbing honeycombs (1#~8#). (**c**) Reflectivity of the honeycomb sandwich composites fabricated from the wave-absorbing honeycomb 1#~8#.

The honeycomb structure is the core functional layer of the wave-absorbing HSC, whose dielectric property and thickness show significant influence on radar stealth performance of the composite. Wave-absorbing honeycombs were fabricated by repeatedly immersing the Nomex honeycomb in a dispersion of conductive carbon black mixed with bismaleimides resin and acetone for several cycles to reach a predetermined weight increment, and finally cured at an elevated temperature in the oven. The Nomex honeycomb is a kind of wave-transmitting material with small dielectric constant (*ε*_r_′ = 1.1) and low loss tangent (tan *δ* = 0.003). Conductive carbon black can attenuate the radar wave through electronic polarization, interfacial polarization and other effects, during which electromagnetic energy is converted into conduction current and finally dissipated into heat. The effective dielectric constant and loss tangent of honeycomb can be promoted by increasing the loading amount of wave-absorbing agent. Figure 3a,b respectively show the real part and imaginary part of the complex relative permittivity of the eight wave-absorbing honeycombs (1#~8#) used for this study. In the frequency range from 1 GHz to 2 GHz, *ε*_r_′ of the honeycombs show the order of 1# < 2# < 3# < 4# ≈ 6# < 5# < 7# < 8# and *ε*_r_″ show the order of 1# < 2# < 3# < 4# < 5# < 6# < 7# < 8#. The reflectivity of the HSC with honeycombs 1#~8# was measured in the frequency range of 1–2 GHz, and the results are shown in Figure 3c. The thickness of wave-absorbing honeycomb is fixed at 30 mm. The results demonstrate that, by increasing the dielectric properties (*ε*_r_′ and *ε*_r_″) of honeycomb, the wave-absorbing performance of HSC gradually increases at first, while rapidly decreasing when complex permittivity exceeds a certain level. The effect of the dielectric property of honeycomb on wave-absorbing performance originated from the following mechanisms. When the real part and imaginary part of permittivity are both small (e.g., 1#~5#), the impedance difference between the wave-transmitting skin and the honeycomb is relatively low, and the electromagnetic wave can effectively pass through their interface and enter into the honeycomb. In this condition, the increasing of *ε*_r_″ enhances the attenuation rate and absorption ratio of the radar wave in the honeycomb structure, which contributes to decrease in the reflectivity of HSC. However, when the dielectric property of honeycomb is sufficiently large (e.g., 7# and 8#), impedance mismatching between the skin and honeycomb leads to considerable reflection of the incident radar wave on the interface, which decreases energy transmission efficiency and weakens wave-absorbing performance of the HSC. Limited by the above reasons, even for honeycomb 6#, which has the best performance among the overall samples, reflectivity in 1–1.3 GHz is still larger than −10 dB, which is unsatisfactory in satisfying the demand for high-performance radar-stealth in the low frequency band. Moreover, wave-absorbing honeycomb with high dielectric properties loads a large amount of the wave-absorbing agent and shows a large density (Table 1), which significantly increases the total weight of the composite structure. Consequently, increasing the dielectric properties of honeycomb is of limited effect in improving low-frequency wave-absorbing performance of the composites in line with the demand for a light weight structure.

#### 3.1.3. Effect of the Thickness of Wave-Absorbing Honeycomb

**Figure 4:**
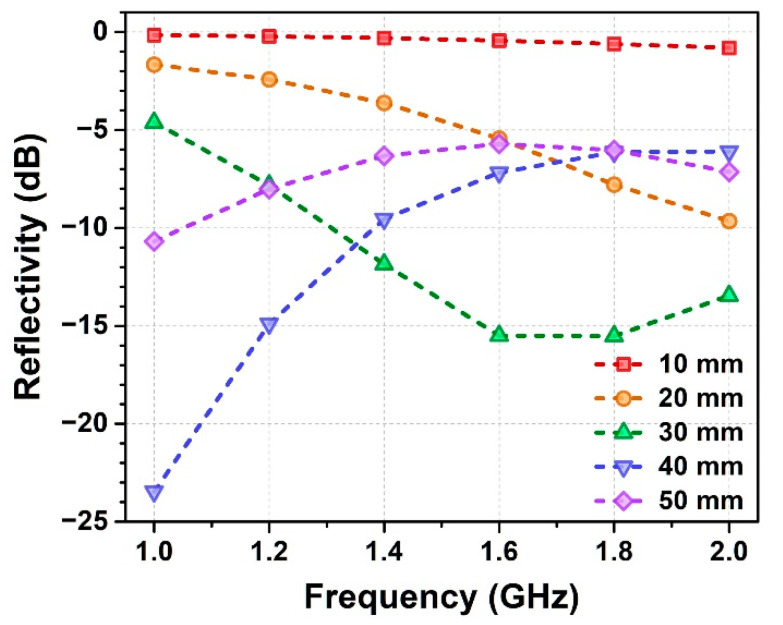
Reflectivity of HSC-6# with different thicknesses of the wave-absorbing honeycomb.

As the low-frequency radar wave has a long wavelength and large penetration depth, the thickness of wave-absorbing honeycomb also affects the wave-absorbing property of HSC to a large extent. The influence of honeycomb thickness was investigated using honeycomb 6#, which exhibits optimal dielectric property (Figure 3c) in the low frequency range compared with the others. Figure 4 shows the reflectivity of HSC with the thickness of honeycomb (6#) ranging from 10 mm to 50 mm. It demonstrates that the honeycombs with moderate thickness of 30 mm and 40 mm possess the best wave-absorbing performances among the samples, where the averaged reflectivity of the corresponding HSC is respectively −11.5 dB and −11.2 dB in the frequency band of 1–2 GHz. For the fixed dielectric property, the honeycombs with lower thickness (e.g., 10 mm and 20 mm) could not sufficiently attenuate the incident radar wave in the transmission length. In this case, increasing honeycomb thickness is beneficial to enhance wave-absorbing performance. However, when honeycomb thickness reaches a much larger level (e.g., 50 mm), the resonant absorption peak of honeycomb moves to a lower frequency region (*f* < 1 GHz), which weakens its wave-absorption ability in the band of interest (1–2 GHz). By balancing these two opposite factors and considering the demand for a light-weight wave-absorbing composite, the honeycomb with moderate thickness of 30 mm was chosen to fabricate the wave-absorbing MHSC in the following sections.

### 3.2. Wave-Absorbing Properties of MHSC

According to the above discussion, increasing the dielectric property (Figure 3) and increasing the thickness (Figure 4) of honeycomb both have limited effect in improving the wave-absorbing property of HSC in the low-frequency band. To solve this problem, we designed a type of MHSC that contains metamaterial structural units with unique electromagnetic response in the frequency range of 1–2 GHz. MHSC was fabricated by incorporating a PEEK layer loading the periodic metal arrays into the conventional HSC structure, which is schematically illustrated in Figure 1. The PEEK film acts as the supporting substrate of the periodic metal units and guarantees position accuracy in the pattern-transfer process. Moreover, during the curing step, the PEEK film can be dissolved in the resin matrix and fused into wave-transmitting skin, which prevents mechanical property loss brought about by the redundant film layer.

**Figure 5:**
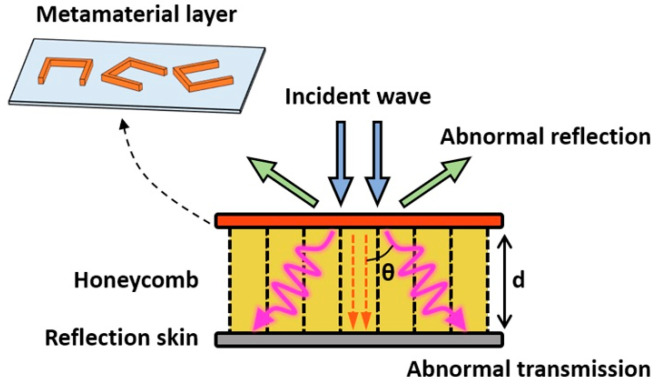
Schematic illustration of the wave-absorbing mechanism of metamaterial/honeycomb sandwich composites (MHSCs).

As shown in Figure 5, the metamaterial layer consists of open square metal rings with subwavelength sizes, which were designed on the basis of the phase gradient principle [45]. These anisotropic metal units can effectively resonate in the electromagnetic field under the specific wavelength, inducing a phase shift to the incident radar wave and producing a phase gradient on the meta-surface. In consequence, when the radar wave is incident on to the metamaterial units, both the reflected wave and the transmitted wave will be deflected with an abnormal reflection/refraction angle. The mechanism is schematically shown in Figure 5. On one hand, abnormal reflection can make the reflected wave deviate from the incident direction, which changes the direction and shape of the reflected lobe and reduces the RCS (Radar Cross Section) of the target. On the other hand, the abnormal transmission can increase the propagation distance (i.e., the effective thickness) of the radar wave in the honeycomb, which can significantly enhance electromagnetic loss and improve the wave-absorbing property of the composites in the low-frequency band.

#### 3.2.1. Properties of MHSC with Wave-Transmitting Honeycomb

**Figure 6:**
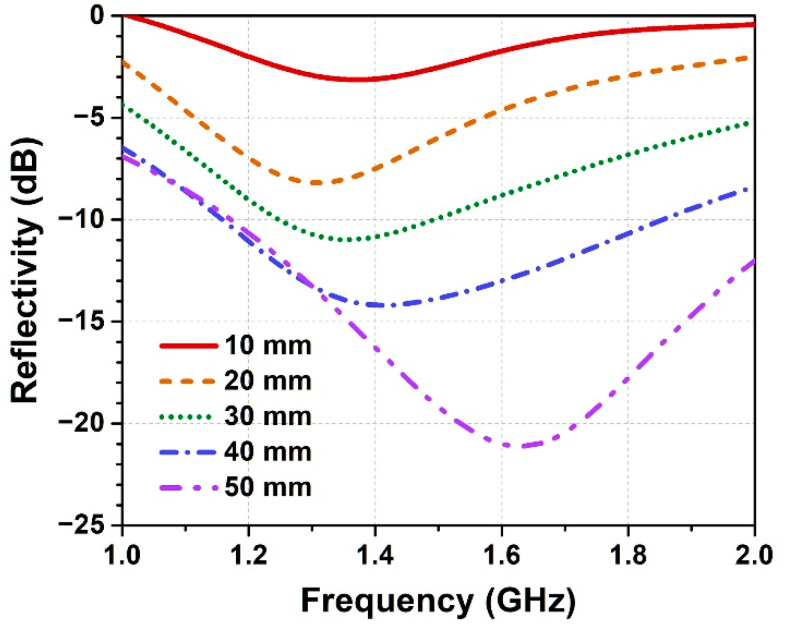
Reflectivity of the MHSC with the different thicknesses of the wave-transmitting Nomex honeycomb.

Firstly, the effect of metamaterial units on the radar stealth performance of composites was investigated using the wave-transparent Nomex honeycomb with different thicknesses ranging from 10 mm to 50 mm. The thickness of the wave-transmitting skin of MHSC was fixed at 1 mm. As shown in Figure 6, increasing the thickness of Nomex honeycomb can significantly decrease the reflectivity of HSCM in the frequency range of 1–2 GHz. This phenomenon is ascribed to the abnormal transmission of the electromagnetic wave on the metamaterial surface. As demonstrated in Figure 5, the effective thickness of the honeycomb (*d*/cos(*θ*), propagation distance of the refracted wave) shows positive correlation with its actual thickness *d*. Consequently, for a certain electromagnetic response (cos(*θ*)) of metamaterial units, a relatively large thickness (*d*) of honeycomb is conducive to fully utilize the radar stealth function of the metamaterials in MHSC. According to Figure 6, when the thickness of Nomex honeycomb is larger than 30 mm, the composite with metamaterial shows relatively small reflectivity (less than −5 dB) in the band. However, the wave-transparent Nomex honeycomb without loading absorbing agents has low dielectric loss and cannot effectively attenuate the electromagnetic wave in the high frequency range (e.g., 8–18 GHz). Consequently, in the next section, wave-absorbing honeycombs with different dielectric constants and dielectric loss were complexed with metamaterial structure to fabricate MHSC, in order to realize more efficient wave-absorbing property in the low frequency range and balanced performance in a wide-frequency range.

#### 3.2.2. Properties of MHSC with Wave-Absorbing Honeycomb

**Figure 7:**
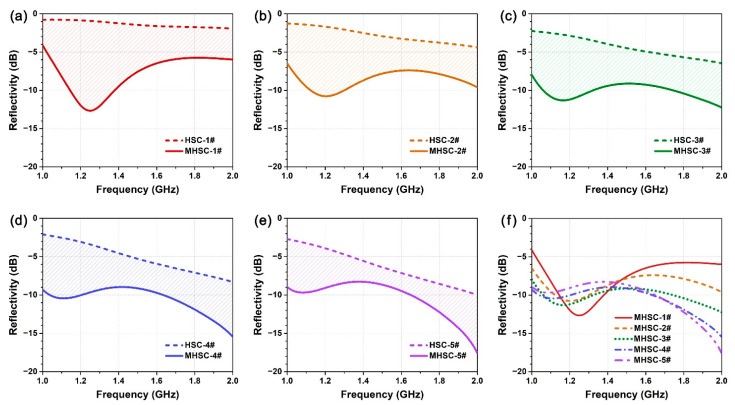
(**a**–**e**) Reflectivity of HSC (dash line) and MHSC (solid line) in the low frequency range (1–2 GHz). The wave-absorbing honeycomb is respectively (**a**) 1#, (**b**) 2#, (**c**) 3#, (**d**) 4# and (**e**) 5#, whose dielectric properties show an ascending order of 1# < 2# < 3# < 4# < 5#. The shadow area in each figure represents the reduction of reflectivity by inserting the metamaterial units in the honeycomb sandwich composite. (**f**) Comparison of the wave-absorbing performance of MHSC fabricated from the honeycomb with different dielectric properties.

Figure 7a–e show the reflectivity curves of five different MHSCs (colored solid lines) in the frequency band of 1–2 GHz, which were compared with corresponding HSCs without metamaterials (black dash lines). The HSCs and MHSCs were fabricated from wave-absorbing honeycombs (1#~5#) with different dielectric properties (see Figure 3a,b) and with a fixed thickness of 30 mm. The results illustrate that, for the five honeycomb samples, incorporating the metamaterial layer in the composite can decrease reflectivity in the low frequency band to a large extent. In fact, the metamaterial designed in this paper is a kind of subwavelength anisotropic structure. Due to the distinct phase-frequency response of the resonant units with different rotational orientation, the metamaterial can generate a gradual phase distribution (i.e., phase gradient) for the reflected and transmitted waves [44,45,46]. This unique electromagnetic effect equivalently changes the shape and direction of the reflected lobe and increases the effective thickness of the honeycomb absorber, which significantly enhances the low-frequency radar stealth performance of the composites. However, as revealed in Figure 7f, the wave-absorbing performance of MHSC is still correlated with the dielectric property of the wave-absorbing honeycomb in a complex manner. To quantitatively evaluate this issue, the averaged reflectivity (*R*_a_) of the HSC and MHSC in the frequency range of 1–2 GHz is extracted from the curves and listed in Table 2. HSC without metamaterial increasing the dielectric property of the honeycomb (1#~5#) within a certain range can significantly enhance radar-stealth performance, where *R*_a_ is decreased from −1.35 dB to −6.29 dB from 1# to 5#. This effect is attributed to the more intensified wave absorption ability of the honeycomb with the larger dielectric loss. By contrast, when incorporated with metamaterials, it is the MHSC-4# with the moderate dielectric property of honeycomb that exhibits the best wave-absorbing performance (*R*_a_ = −10.59 dB) among the five MHSC samples. As shown in the second row of Table 2, for honeycombs 1#, 2#, 3#, 4# and 5# with increased dielectric property, averaged reflectivity increases first and then decreases. In other words, the wave-absorbing performance of the metamaterial units is strongly restricted by the dielectric properties of the honeycomb structure. The value of (*R*_a,HSC_ − *R*_a,MHSC_) is the reflection reduction of MHSC compared with HSC, which represents the enhancing effect of the radar stealth performance contributed by the metamaterial layer in the composite. As shown in the last row of Table 2, this value is negatively correlated with the dielectric property of the wave-absorbing honeycomb. Specifically, for the wave-transmitting Nomex honeycomb with the lowest dielectric constant and lowest dielectric loss, the metamaterial decreases the averaged reflectivity for 8.14 dB in the low frequency band, while the value is only 4.09 dB for the wave-absorbing honeycomb 5# with the largest dielectric property among the samples. The mechanism is that, although the honeycomb with larger dielectric loss exhibits better wave-absorbing property, it will also show more significant interference on the electromagnetic response of the metamaterial layer. To achieve optimal radar-stealth performance in the low frequency band, the metamaterial units should be complexed with the wave-absorbing honeycomb that exhibits well-matched dielectric properties.

**Figure 8:**
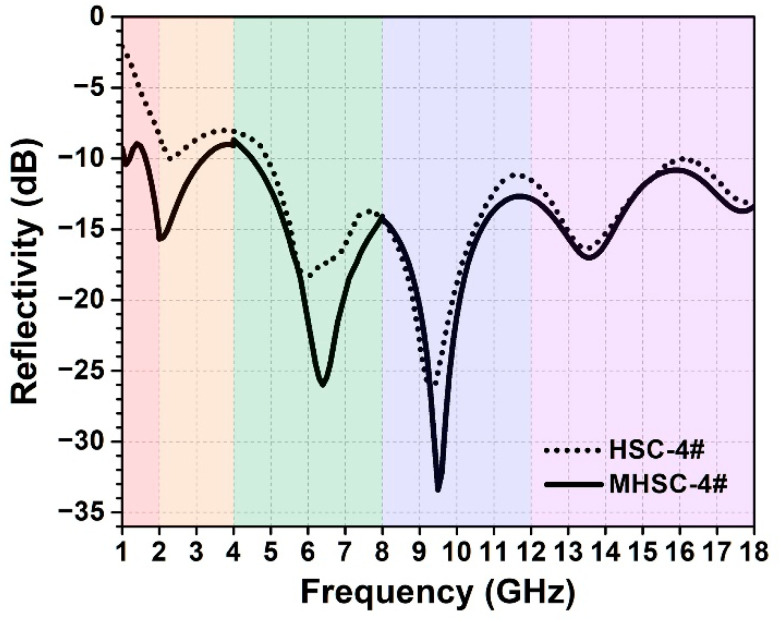
Wide-band wave-absorbing performance of the MHSC (solid line) and HSC (dot line) in the frequency range from 1 GHz to 18 GHz. Both the composites used the wave-absorbing honeycomb 4# with a thickness of 30 mm. From left to right, the five regions with the colors of red, yellow, green, blue and purple respectively represent frequency bands of 1–2 GHz, 2–4 GHz, 4–8 GHz, 8–12 GHz and 12–18 GHz.

According to the above investigation, the fabricated honeycomb sandwich composite with metamaterial (e.g., MHSC-4#) can realize excellent low-frequency wave-absorbing performance with averaged reflectivity as low as −10.59 dB in the band of 1–2 GHz. Compared with HSC-4# with the same thickness of wave-transmitting skin (1 mm) and wave-absorbing honeycomb (30 mm), incorporating the phase-gradient metamaterial into the composite can significantly enhance the wave-absorbing performance for 5.43 dB, with an increment larger than 100%. Meanwhile, the thin metamaterial layer with a surface density of only 0.19 kg/m^2^ (much lower than that of the wave-absorbing honeycombs, see Table 1) shows no obvious weight-increase to the total structure. Moreover, under dielectric match between the honeycomb and metamaterial, the MHSC also possesses a more satisfactory and enhanced wide-band wave-absorbing property than the corresponding HSC. The reflectivity of MHSC-4# and HSC-4# in the frequency range of 1–18 GHz was also characterized, and the curves are shown in Figure 8. According to the results, averaged reflectivity in the frequency band of 1–4 GHz is −11.18 dB for MHSC-4# but only −7.51 dB for HSC-4#. The −10 dB bandwidth in the frequency band of 1–8 GHz is 5.4 GHz for MHSC-4# but only 3.16 GHz for HSC-4#. Moreover, over an extremely wide frequency range of 1–18 GHz, the averaged reflectivity of the HSCM-4# is lower than −14.8 dB, with the lowest reflectivity of −33.4 dB at the frequency of 9.5 GHz. These results indicate that, when matched with a suitable honeycomb, incorporation of the phase-gradient metamaterial layer can significantly enhance the wave-absorbing properties of the composites in the low frequency band while effectively maintaining original wave-absorbing performance in the high frequency range. It is strongly believed that the MHSCs reported in this study show promising potential to be applied as high-performance radar stealth composite materials.

## 4. Conclusions

In summary, we have fabricated a new type of metamaterial/honeycomb sandwich composite (MHSC) and systematically investigated wave-absorbing performance in the low frequency band of 1–2 GHz. It is proved that, simply increasing dielectric properties and heights of the wave-absorbing honeycomb both show a limited effect in improving low-frequency radar stealth performance of the conventional HSC, which is ascribed to the long wavelength of the electromagnetic wave in the band. This challenging problem is successfully solved by incorporating a phase-gradient metamaterial layer in the composite via a pattern-transfer method and optimizing the designing parameters of the fabricated MHSC. When the height of the wave-absorbing honeycomb and the height of the wave-transmitting skin are respectively 30 mm and 1 mm, the fabricated MHSC can possess averaged reflectivity less than −10 dB in the frequency band of 1–2 GHz, whose wave-absorbing performance is promoted for larger than 5 dB compared with the corresponding HSC. It is discovered that enhancement of radar stealth performance in the low frequency band contributed by the metamaterial layer shows negative correlation with dielectric properties of the wave-absorbing honeycomb, which is caused by coupling interference between the two response structures. The optimal low-frequency wave-absorbing property of MHSC is achieved by elaborately balancing the dielectric loss of the honeycomb and the electromagnetic response of the metamaterial units. Under rational design, the MHSC loaded with a thin and light-weight metamaterial layer not only possesses more excellent low-frequency (1–2 GHz) wave-absorbing properties compared with the conventional HSC but also shows more satisfactory radar stealth performance over a wide frequency range of 1–18 GHz. This study both proposes an effective methodology to enhance the low-frequency wave-absorbing performance of conventional HSCs and provides deep insight into electromagnetic coupling between the honeycomb absorber and metamaterials. It is highly expected that the discoveries and design methods of this work can be extended to fabricate light-weight and wide-band MAMs based on metamaterials and other composite structures, such as laminates, foams, fabrics, aerogels, and so on.

## Figures and Tables

**Table 1:** Density of the wave-absorbing honeycomb.

Wave-Absorbing Honeycomb	1#	2#	3#	4#	5#	6#	7#	8#
Density (g/cm^3^)	0.06	0.07	0.08	0.09	0.1	0.15	0.2	0.3
Surface density (kg/m^2^) ^1^	1.8	2.1	2.4	2.7	3	4.5	6	9

^1^ The surface density was calculated for the honeycomb with the thickness of 30 mm.

**Table 2:** Averaged reflectivity of HSC and MHSC with different honeycombs in the frequency band of 1–2 GHz.

Honeycomb	Nomex	1#	2#	3#	4#	5#
*R*_a,HSC_	0	−1.35	−2.80	−4.35	−5.16	−6.29
*R*_a,MHSC_	−8.14	−7.85	−8.64	−10.18	−10.59	−10.38
*R*_a,HSC_ − *R*_a,MHSC_	8.14	6.50	5.84	5.83	5.43	4.09

## Data Availability

Not applicable.

## Supplementary Materials

The following supporting information can be downloaded at: https://www.mdpi.com/article/10.3390/polym14071424/s1, Figure S1: Photograph of the fabricated wave-transmitting skin containing metamaterial units; Figure S2: Schematic illustration about the structure and wave-absorbing mechanism of the honeycomb sandwich composites (HSC).
