# Supplementary material for: Study on Microwave Absorption Performance Enhancement of Metamaterial/Honeycomb Sandwich Composites in the Low Frequency Band
Source: Polymers (Basel). 2022 Mar 31;14(7):1424. doi: 10.3390/polym14071424 (PMC9002943; doi:10.3390/polym14071424)
Supplement: Supplementary file 1 [file polymers-14-01424-s001.zip › polymers-1625074-supplementary.pdf]

Supplementary Materials

Study on microwave absorption performance enhancement of metamaterial/honeycomb sandwich composites in the low frequency band

Songming Li [1,2], Hao Huang [1], Sibao Wu [1], Jiafu Wang [3], Haijun Lu [1,2] and Liying Xing [1,2,]*

1. Composite Technology Center, AVIC Beijing Aeronautical Manufacturing Technology Research Institute, Beijing 101300, China.
2. National Key Laboratory of Advanced Composites, AECC Beijing Institute of Aeronautical Materials, Beijing 100095, China.
3. Department of Basic Sciences, Air Force Engineering University, Xi’ an 710051, China.

** Corresponding author. Email: vcd4321@sina.com*

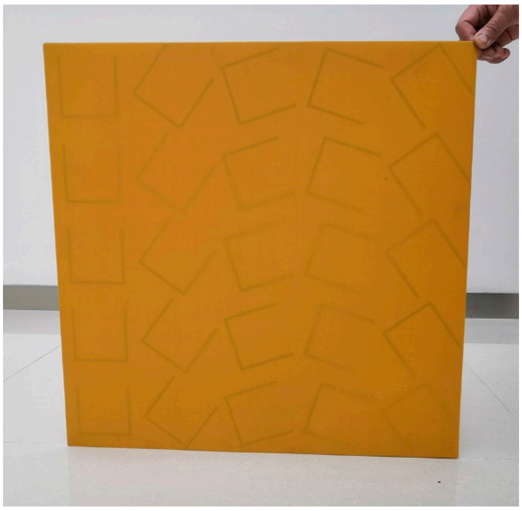

Figure S1. Photograph of the fabricated wave-transmitting skin containing metamaterial units.

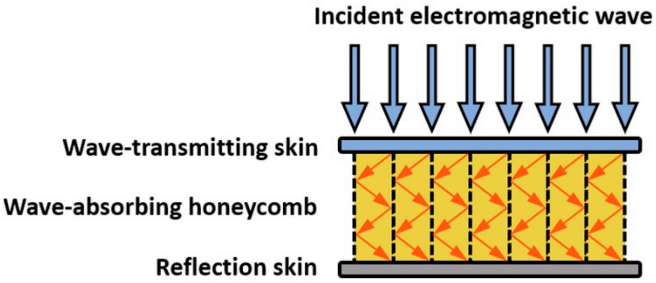

Figure S2. Schematic illustration about the structure and wave-absorbing mechanism of the honeycomb sandwich composite (HSC).
